# Cytomegalovirus Viremia Is Associated With Mortality Among Children With HIV Starting Antiretroviral Therapy in Sub-Saharan Africa

**DOI:** 10.1093/cid/ciag354

**Published:** 2026-06-09

**Authors:** Temitope Fisayo, Ceri Evans, Kuda Mutasa, Dagmar Alber, Moira J Spyer, Sandra Rukobo, Mutsa Bwakura-Dangarembizi, Victor Musiime, Kusum J Nathoo, Ade Kekitiinwa, Cissy Kityo, Diana M Gibb, Nigel Klein, A Sarah Walker, Andrew J Prendergast

**Affiliations:** Blizard Institute, Queen Mary University of London, London, United Kingdom; Zvitambo Institute for Maternal and Child Health Research, Harare, Zimbabwe; Clinical Infection, Microbiology and Immunology, University of Liverpool, Liverpool, United Kingdom; Zvitambo Institute for Maternal and Child Health Research, Harare, Zimbabwe; Infection, Immunity and Inflammation Research and Teaching Department, UCL Great Ormond Street Institute of Child Health, London, United Kingdom; MRC Clinical Trials Unit at UCL, University College London, London, United Kingdom; Zvitambo Institute for Maternal and Child Health Research, Harare, Zimbabwe; Faculty of Medicine and Health Sciences, University of Zimbabwe, Harare, Zimbabwe; Department of Paediatrics and Child Health, Makerere University, Kampala, Uganda; Faculty of Medicine and Health Sciences, University of Zimbabwe, Harare, Zimbabwe; Baylor College of Medicine Children's Foundation, Kampala, Uganda; Joint Clinical Research Centre, Kampala, Uganda; MRC Clinical Trials Unit at UCL, University College London, London, United Kingdom; Infection, Immunity and Inflammation Research and Teaching Department, UCL Great Ormond Street Institute of Child Health, London, United Kingdom; MRC Clinical Trials Unit at UCL, University College London, London, United Kingdom; Blizard Institute, Queen Mary University of London, London, United Kingdom; Zvitambo Institute for Maternal and Child Health Research, Harare, Zimbabwe

**Keywords:** HIV, CMV, mortality, children, sub-Saharan Africa

## Abstract

**Background:**

Cytomegalovirus (CMV) co-infection is associated with mortality in adults with human immunodeficiency virus (HIV) infection, but whether CMV is associated with mortality in children with HIV remains uncertain.

**Methods:**

In 498 children (median age 6.3 years, interquartile range 2.3–9.6) enrolled in the ARROW trial (ISRCTN24791884) in Uganda (336/498) and Zimbabwe (162/498) selected using a case-cohort design, CMV was quantified using real-time polymerase chain reaction at initiation of non-nucleotide reverse transcriptase inhibitor-based antiretroviral therapy (ART), 12 weeks post-ART, and 84 weeks post-ART. Associations between baseline CMV viremia and mortality were evaluated using multivariable models, adjusting for baseline HIV RNA, CD4^+^ percentage, and IL-6 concentrations.

**Results:**

Baseline CMV viremia was associated with mortality, with relationships differing by country and assay. In Zimbabwe (assay limit of detection 20 copies/mL), 119/162 (73%) children had detectable CMV, and each log_10_ higher CMV viral load was associated with over 2-fold higher mortality (adjusted hazard ratio [aHR] = 2.74; 95% CI 1.57–4.77). In Uganda (assay limit of detection 120 copies/mL), 89/336 (26%) children had detectable CMV viremia, which was associated with 3-fold higher mortality compared to undetectable CMV (aHR = 3.15; 95% CI 1.11–8.93). In a subset of 48 children with immunophenotyping data, we found no evidence that CMV was associated with immune activation.

**Conclusions:**

Cytomegalovirus viremia is independently associated with mortality in children with HIV starting ART in sub-Saharan Africa. Future studies should define the underlying mechanisms and evaluate whether suppressing CMV viremia reduces mortality in children with HIV.

## Teaser

Among 498 children with HIV in Uganda and Zimbabwe (median age 6.3 years), cytomegalovirus (CMV) viremia at initiation of antiretroviral therapy was independently associated with increased mortality. Deaths were most commonly due to pneumonia and sepsis, not CMV end-organ disease.

**(See the Editorial Commentary by Ellis and Jarvis on pages e816–8.)**

Despite progress in preventing vertical human immunodeficiency virus (HIV-1) transmission, approximately 130 000 children are diagnosed with HIV annually, 80% of whom live in sub-Saharan Africa [1]. Compared with adults, children with HIV are less likely to be taking antiretroviral therapy (ART): Globally, around 57% of children with HIV below 15 years are on ART [2]. Although immediate treatment initiation is recommended [3], the median age at starting ART among children in sub-Saharan Africa is 19 months [4]. A substantial number of children therefore start ART with advanced HIV, with a high risk of mortality in the first few months after initiating ART [5].

Cytomegalovirus (CMV) is a common co-infection among children with HIV. In sub-Saharan Africa, CMV is typically acquired in infancy through breastfeeding or household contact, in contrast to high-income settings where only 50% of children have acquired CMV by adolescence [6–8]. While several studies have shown that CMV co-infection is associated with increased mortality in adults with HIV, the effects of CMV in children with HIV are incompletely understood [9–11]. Cytomegalovirus viremia is associated with lower CD4^+^ counts in both adults and children [11, 12], and CMV drives CD8^+^ T-cell activation and apoptosis in infants with HIV [13]. Cytomegalovirus is a co-factor in disease progression in adults with HIV irrespective of ART [10, 14]; however, the association between CMV viremia at ART initiation and subsequent mortality is underexplored in children. In this study, we investigated the impact of CMV viremia on mortality in a cohort of children commencing ART in Uganda and Zimbabwe.

## METHODS

Participants were enrolled in the ARROW trial (ISRCTN24791884), which has been previously described [15]. In brief, Ugandan and Zimbabwean children and adolescents with HIV, who were previously untreated, aged 3 months–17 years, and met criteria for ART in 2006 World Health Organization guidelines [16], were eligible. Children were recruited from urban (Joint Clinical Research Centre [JCRC] and Paediatric Infectious Disease Clinic [PIDC], both in Kampala) and nonurban sites (Entebbe) in Uganda and from urban Harare in Zimbabwe. Children were randomized 1:1:1 to start ART with continuous lamivudine + abacavir + non-nucleoside reverse transcriptase inhibitor (NNRTI) (Arm-A), or induction maintenance with 4-drug lamivudine + abacavir + NNRTI + zidovudine for 36 weeks, followed by lamivudine + abacavir + NNRTI (Arm-B) or lamivudine + abacavir + zidovudine (Arm-C). The NNRTI (nevirapine/efavirenz) was chosen by clinicians based on availability. Children were simultaneously randomized using a factorial design 1:1 to clinically driven monitoring versus laboratory plus clinical monitoring.

A subgroup of 600 children was selected for inclusion in a nested case-cohort study, as previously described [17], with the overarching aim of characterizing virological and immunological responses in children initiating ART in Africa. Cases (N = 115) were children who died or experienced new/recurrent World Health Organization (WHO) stage 4 events [18] during follow-up (to trial closure, median 4-year follow-up) or were immunological nonresponders (CD4% ≤15% through 3 years on first-line ART, allowing a single CD4^+^ measurement >15%). Controls (N = 485) comprised children in a longitudinal immunology substudy (N = 316; those providing additional consent and recruited from May to November 2008 but otherwise unselected) and a random 23% sample of all remaining nonsubstudy children (to complete the sample size of 600). Causes of death among cases were established by an independent expert review committee, who reviewed a clinical narrative and available laboratory and radiology results. Deaths in the current study were categorized using the primary (ie cause 1a, immediate) cause of death.

Blood sampling and plasma storage at −80°C occurred prior to ART initiation (either at enrollment or at screening, which was maximum 30 days pre-enrollment) and at 12 and 84 weeks post-ART initiation in all children selected for the case-cohort design. Cryopreserved plasma was used to measure inflammatory biomarkers (C-reactive protein (CRP), tumor necrosis factor alpha (TNF-α), interleukin-6 (IL-6), and soluble CD14 (sCD14)) and IL-7 by ELISA (R&D Systems, Oxford, UK), and HIV RNA by Abbott m2000sp/rt (Uganda) or Roche COBAS Amplicor Monitor v1.5 (Zimbabwe), as previously described [17]. Total CD4^+^ and CD8^+^ T-cells were measured in real time. In Uganda, children in the immunology substudy had whole blood immunophenotyping using anti-CD4-PerCP (Becton Dickinson, BD), anti-CD45RA-APC (Caltag Medsystems), anti-CD31-PE (eBioscience), and either anti-Ki67-FITC (BD; after nuclear membrane permeabilization) or anti-HLA-DR-FITC (BD), with data acquired on a BD FACSCalibur flow cytometer. Analysis was undertaken using Cellquest (BD), to define the following subpopulations as a percentage of total CD4: CD45RA^+^ (naive), CD45RA^+^ CD31^+^ (recent thymic emigrants), HLA-DR^+^ (activated), and Ki67^+^ (proliferating) cells.

### Cytomegalovirus DNA polymerase chain reaction

All children selected for the case-cohort design with sufficient remaining plasma prior to ART initiation had CMV quantified by real-time polymerase chain reaction (PCR); where there were available samples, longitudinal CMV DNA PCR was also undertaken at 12 and 84 weeks post-ART. The PCR assay differed by country. In Zimbabwe, viral nucleic acid was extracted from 500 µL plasma, using the Abbott RealTime CMV kit following a manual sample preparation method. Each extraction included positive and negative controls. The Abbott RealTime CMV assay was used to quantitate CMV DNA on the Abbott m2000rt platform in Harare. The upper and lower limits of quantification for this assay in plasma are 100 million copies/mL and 20 copies/mL, respectively. Ugandan samples were shipped to the UK on dry ice. Viral nucleic acid was extracted from 100 µL of plasma (or 50 µL if only small plasma volumes remained), using the Quick-DNA 96 kit (Zymo Research). Each extraction included positive and negative controls. An in-house assay was used to quantitate CMV DNA on a CFX96 C1000 Touch (BioRad), using methods previously described [17]. The upper and lower limits of detection for this assay are 20 million copies/mL and 200 copies/mL, respectively. Samples that were detectable but not quantifiable (ie with strongly positive cycles) had values set to 120 copies/mL.

### Statistical Analysis

The primary outcome considered in this secondary analysis of data from the case-cohort was mortality, analyzed as a time-to-event outcome using Cox regression with time zero at ART initiation, censoring at the end of trial follow-up. The other components of the original case definition for selection into the case-cohort (new WHO 4 events/immunological response) were not considered as outcomes because we were interested in estimating associations with mortality as the most important clinical outcome. We therefore did not use sampling weights, but instead adjusted analyses estimating the independent association between mortality and the primary exposures, CMV detection (binary), and CMV viral load (log_10_ transformed), for baseline IL-6, HIV RNA, age, and CD4^+^ percentage as prespecified covariates, and for participation in the longitudinal immunology substudy. We selected IL-6 and CD4% as factors associated with mortality in this cohort [17] and age and HIV RNA because of their strong associations with CMV viremia. Adjusted analyses included all participants, but included interactions between country and CMV variables, given the different assays used. Secondary adjusted analyses considered CMV viremia >1000 copies/mL or >120 copies/mL (the higher of the 2 limits of detection, in the Ugandan assay) as the sole exposure. Unadjusted comparisons used univariable rank-sum and chi-squared tests. To reduce the influence of outliers, measurements were winsorized, with values above the 97.5th percentile set to the 97.5th percentile, and values below the 2.5th percentile set to the 2.5th percentile. To assess independent associations between CMV viremia (continuous variable) at baseline and other baseline covariates, and to estimate CMV viremia at 12 and 84 weeks after ART initiation, we used interval regression, assuming that values below the limit of detection for each assay (log_10_ transformed) lay between the limit and 0.001 copies/mL (−2 on the log_10_ scale). All analyses used STATA version 17.0 (StataCorp, College Station, Texas). All *P* values are 2 sided. The level of statistical significance was set at *P* < .05.

## RESULTS

Overall, 1206 children were enrolled in the ARROW trial [15], of whom 600 were selected into the case-cohort study (see METHODS); among these, 498 (83%) had sufficient plasma to be included in this analysis (104 cases using the original definition for selection, 394 controls; Supplementary Figure 1), comprising 336 (67%) from Uganda and 162 (33%) from Zimbabwe. Overall, participants were median 6.3 years old (interquartile range [IQR] 2.3–9.6) when they started ART with median CD4^+^ percentage 11.4% (IQR 6.9, 17.0); around half were male. Among 104 cases, 46 (n = 17 in Zimbabwe, n = 29 in Uganda) children died. The characteristics of children who died versus survived are detailed in Table 1 (median follow-up 3.7 years [IQR 3.6–4.1]). Characteristics of children in the 2 countries are shown in Supplementary Table 1. Children from Harare were older (median age 7.5 years, IQR 3.9–9.7, vs 5.2 years, IQR 2.2–9.6, in Uganda), with slightly lower CD4^+^ percentage and more advanced WHO stage, but there was no evidence of differences in baseline HIV RNA, CRP, IL-6, or sex.

**Table 1.** Baseline Characteristics of Children Starting NNRTI-Based ART in Uganda and Zimbabwe by Vital Status

Characteristic	Overall (N = 498)	Died (N = 46)	Survived (N = 452)	*P*
Site				.20
Uganda Entebbe (nonurban)	57 (11.5%)	6 (13.0%)	51 (11.3%)	
Uganda JCRC (urban)	142 (28.5%)	8 (17.4%)	134 (29.7%)	
Uganda PIDC (urban)	137 (27.5%)	15 (32.6%)	122 (27.0%)	
Zimbabwe Harare (urban)	162 (32.5%)	17 (37.0%)	145 (32.1%)	
In immunology substudy	256 (51.4%)	8 (17.4%)	248 (54.9%)	<.001
Male sex	239 (47.8%)	24 (52.4%)	215 (47.3%)	.52
Age, years; median (IQR)	6.3 (2.3, 9.6)	7.2 (2.3, 11.0)	6.1 (2.3, 9.6)	.31
HIV RNA, copies/mL; median (IQR)	238 723 (81 090, 641 190)	228 753 (76 330, 1 437 321)	239 789 (78 174, 631 785)	.18
CD4%; median (IQR)	11 (6–17)	6 (2–12)	12 (7–17)	<.001
WHO clinical stage				.02
1	9 (1.8%)	0 (0%)	9 (2.0%)	
2	147 (29.5%)	9 (19.6%)	138 (30.5%)	
3	264 (53.0%)	22 (47.8%)	242 (53.5%)	
4	78 (15.7%)	15 (32.6%)	63 (13.9%)	
Detectable CMV at baseline	208 (41.8%)	31 (67.4%)	177 (39.2%)	<.001
Plasma CRP, mg/L; median (IQR)	4.6 (1.4–14.6)	10.7 (2.4–23.3)	4.1 (1.4–13.3)	.01
Plasma IL-6, pg/mL; median (IQR)	6.2 (4.7–9.5)	8.6 (5.6–15.6)	6.0 (4.7–9.3)	<.001

Abbreviations: JCRC, Joint Clinical Research Centre; PIDC, Paediatric Infectious Disease Clinic, Mulago Hospital.

### Cytomegalovirus Viremia in Children Starting Antiretroviral Therapy

Cytomegalovirus was detected at ART initiation in 206/498 (41%) of children. Children with detectable CMV were younger than those without detectable CMV (median age 5.8 years [IQR 2.1–9.5] vs 7.1 years [IQR 3.7–9.8] *P* = .04), and a greater proportion were female (54% [112/206] vs 44% [127/291], respectively; *P* = .02).

In Uganda (assay limit of detection 120 copies/mL), CMV was detected in 89 (26%) of 336 children (16% [9/57] of children at the Entebbe site, 22% [31/142] at the JCRC site, and 36% [49/137] at the PIDC site). Where detected, median CMV viral loads were 316 copies/mL (IQR 242–399), 1213 copies/mL (IQR 462–7021), and 606 copies/mL (IQR 304–1006), respectively. Thirty-two of 336 children (10%) from Uganda had CMV viral loads ≥1000 copies/mL at ART initiation. Among these 32 children, the median age was 1.8 years (IQR 0.9–3.5); 15/33 (45%) were female, and the median CD4% was 8% (IQR 3%–12%) compared with 5.8 years (2.3–10.2) (*P* < .0001), 165/304 female (54%) (*P* = .46), and median CD4% 12% (7%–19%) (*P* = .005) in those with CMV <1000 copies/mL (including undetectable).

In Zimbabwe (assay limit of detection 20 copies/mL), CMV was detected in 119 (73%) of 162 children; where detected, the median CMV viral load was 163 copies/mL (IQR 67–458). Seventeen of 162 children (10%) from Zimbabwe had CMV viral loads ≥1000 copies/mL at ART initiation. Among these 17 children, the median age was 4.5 years (IQR 1.1–9.8); 9/17 (53%) were female, and the median CD4% was 3% (IQR 1%–10%), compared with 7.6 years (4.7–9.7) (*P* = .15), 70/145 female (48%) (*P* = .80), and 11% median CD4 (6%, 15%) (*P* = .002) in those with CMV <1000 copies/mL (including undetectable).

### Cytomegalovirus Viremia Is Associated With Age, HIV RNA, CD4%, and WHO Stage

Figure 1 illustrates the univariable associations between CMV viremia and HIV RNA (Figure 1*A*), age (Figure 1*B*), CD4% (Figure 1*C*), and WHO stage (Figure 1*D*). In both countries, there was a positive association between baseline HIV RNA and CMV viral load (Spearman's ρ = 0.27; *P* < .001), and a negative correlation between CMV viremia and age (ρ = −0.31; *P* < .001), and between CMV viremia and CD4% (ρ = −0.11; *P* = .012). Participants with more advanced WHO clinical stage had higher CMV viral loads. These associations persisted independently in a multivariable interval regression model (Supplementary Table 2). In those with detectable baseline CMV viremia, CMV viral loads declined after ART initiation (*P* < .001 in both countries at both 12 and 84 weeks vs baseline; Figure 2*A*), with the greatest declines between baseline and 12 weeks (by 92% [95% CI 60%–98%] in Uganda and by 55% [39%–72%] in Harare). The percentages undetectable also rose, to a greater degree in Uganda where the assay threshold was higher (Figure 2*B*).

**Figure 1.**
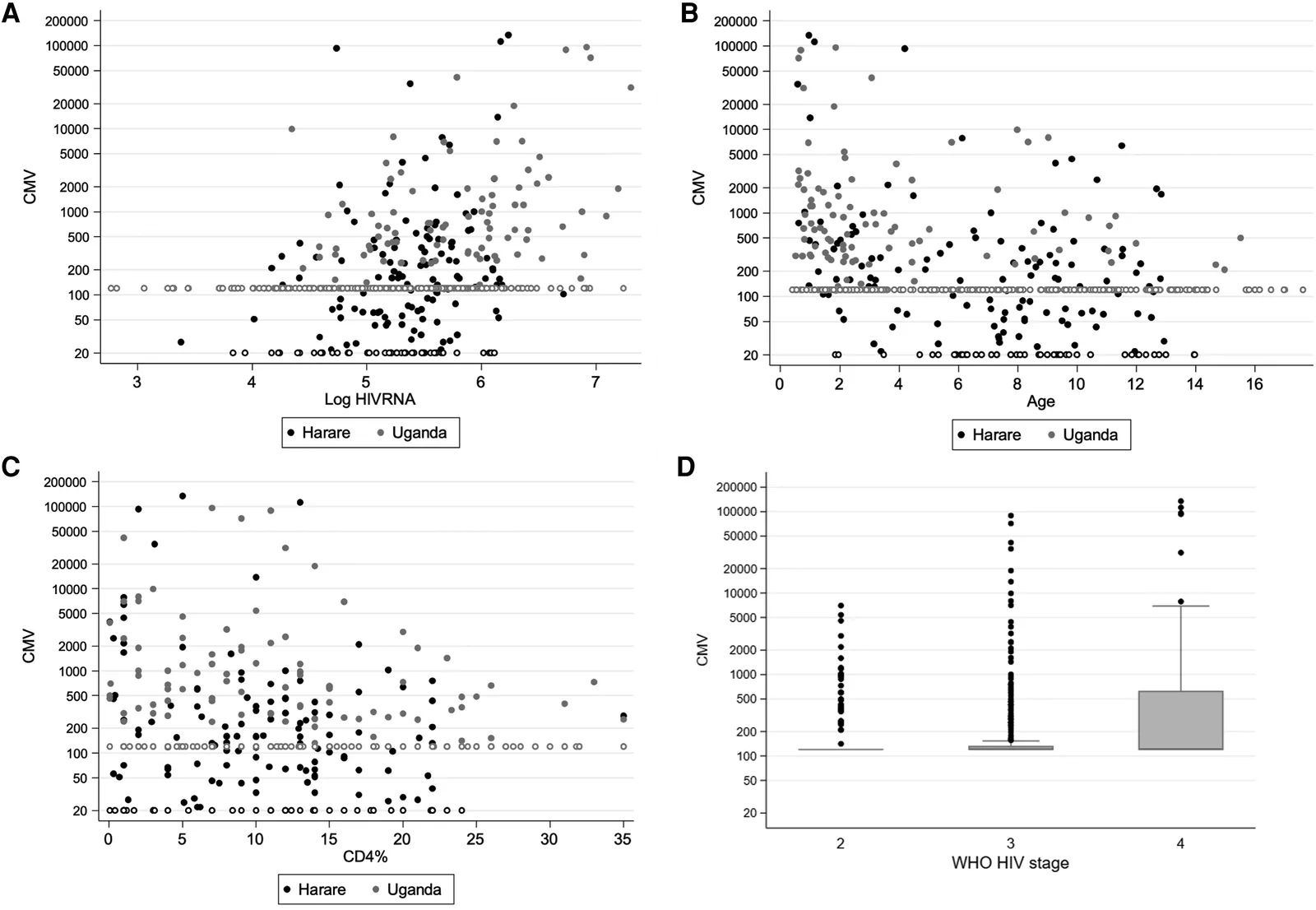
Association between cytomegalovirus (CMV) viremia at antiretroviral therapy (ART) initiation, and (A) HIV RNA, (B) age, (C) CD4%, and (D) WHO stage. Open symbols show values at the limit of detection (20 copies/mL in Zimbabwe, 120 copies/mL in Uganda). In panel *D*, all values <120 are shown at 120 copies/mL.

**Figure 2.**
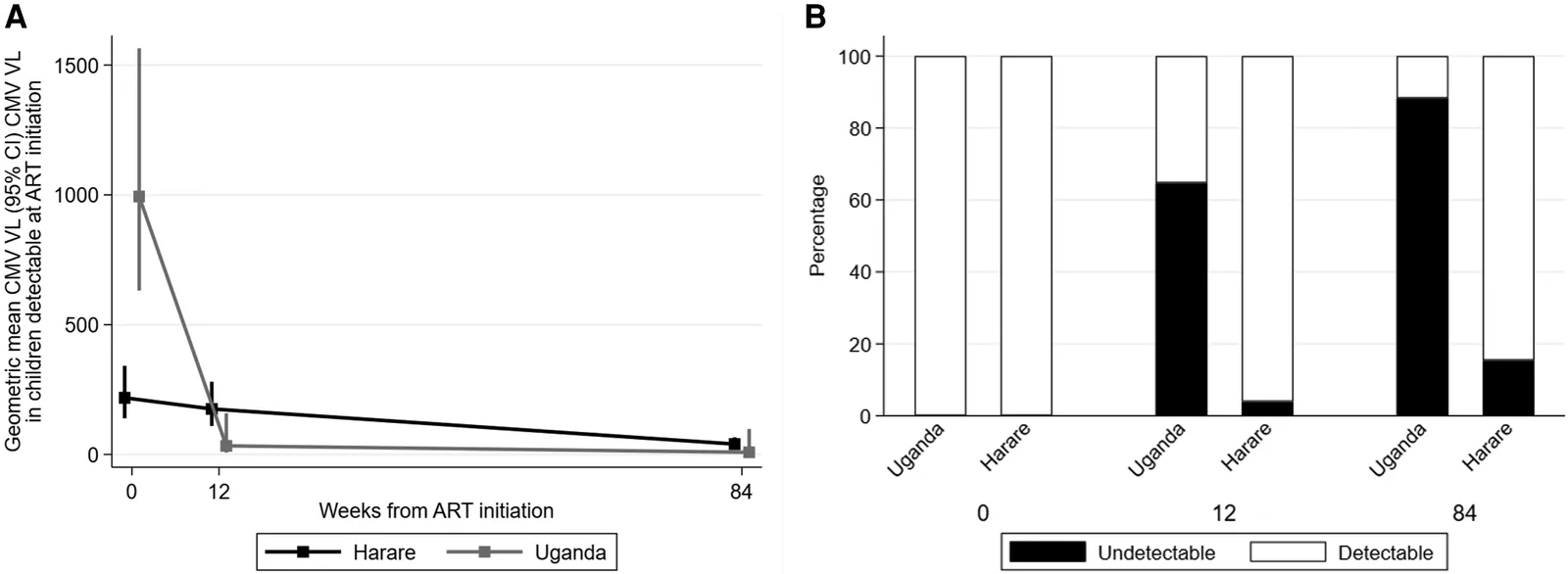
(*A*) Geometric mean cytomegalovirus (CMV) viral load and (*B*) percentage with undetectable CMV viral load following antiretroviral therapy (ART) initiation in those with detectable CMV viremia at ART initiation. Restricted to children in whom follow-up samples were available. *A*, Adjusted for the different limit of detection in the 2 countries using interval regression; see METHODS. *B*, Different assay thresholds in Uganda (120 copies/mL) and Zimbabwe (20 copies/mL.

### Baseline Cytomegalovirus Viremia at Antiretroviral Therapy Initiation Is Associated With Mortality

Cytomegalovirus was associated with mortality in both countries (Table 2). Cytomegalovirus was detected at ART initiation among 31/46 (67%) children who died, compared to 177/455 (39%) children who survived. In Uganda, where 29 (9%) of 336 children died, children with detectable CMV (threshold 120 copies/mL) compared to those without detectable CMV had 3-fold higher mortality (adjusted hazard ratio [aHR] 3.15 (95% CI 1.11–8.93]; *P* = .03); however, there was no evidence that the degree of CMV viremia was associated with mortality (aHR per log_10_ higher where CMV detected 0.86 [95% CI .37–2.02]; *P* = .73). In Zimbabwe, where 17 (10%) of 162 children died, there was no evidence that children with versus without detectable CMV (threshold 20 copies/mL) had higher mortality (aHR 1.77 [95% CI .20–16.1]; *P* = .61); however, there was a strong association between higher baseline CMV viremia and increased mortality, with over 2-fold higher mortality per log_10_ higher CMV (aHR 2.74 [95% CI 1.57–4.77]; *P* < .001). Secondary analyses combining children from both countries, which dichotomized CMV viral load at 120 copies/mL or 1000 copies/mL, showed similar associations (Table 2). Associations were similar or slightly stronger after excluding IL-6 from the models (Supplementary Table 3).

**Table 2.** Associations Between Mortality and CMV Viremia Among Children Starting NNRTI-Based ART in Uganda and Zimbabwe

	Unadjusted HR (95% CI)	*P*	Adjusted^a^ HR (95% CI)	*P*	Heterogeneity in Effect by Country
Primary analysis					
Detectable versus not detectable using country-specific assay thresholds					
Uganda	3.01 (1.09, 8.25)	.03	3.15 (1.11, 8.93)	.03	…
Zimbabwe	2.01 (.23, 17.3)	.52	1.77 (.20, 16.1)	.61	*P* = .64
Per log_10_ higher CMV (if detectable using country-specific assay thresholds)					
Uganda	1.03 (.48, 2.20)	.95	.86 (.37, 2.02)	.73	…
Zimbabwe	2.37 (1.52, 3.68)	<.001	2.74 (1.57, 4.77)	<.001	*P* = .02
Secondary analyses					
Above versus below 120 copies/mL					
Uganda	3.08 (1.49, 6.38)	.002	2.43 (1.08, 5.46)	.03	…
Zimbabwe	2.38 (.88, 6.43)	.09	2.09 (.76, 5.74)	.16	*P* = .81
Combined	2.85 (1.59, 5.10)	<.001	2.30 (1.22, 4.34)	.01	
Above versus below 1000 copies/mL					
Uganda	2.00 (.76, 5.23)	.16	1.32 (.44, 3.93)	.62	…
Zimbabwe	13.9 (5.33, 36.3)	<.001	9.94 (3.69, 26.8)	<.001	*P* = .005
Combined	4.59 (2.45, 8.60)	<.001	3.58 (1.69, 7.57)	.001	…

Abbreviation: HR, hazard ratio.

^a^One Cox model fitted for all children, adjusted for participation in the immunology substudy, CD4, HIV RNA, age, and plasma IL-6 concentration at ART initiation and including interaction between CMV variables and country. Evidence that the effect of CMV viremia (log_10_) varied across country in the main model (interaction *P* = .02). No evidence that effect of other factors varied by country in the main model (interaction *P* > .05).

Taken together, we found a relationship between baseline CMV viremia and mortality after ART initiation in both Uganda and Zimbabwe. In Zimbabwe, where the assay was more sensitive, CMV viral load was associated with mortality; in Uganda, where the assay was less sensitive, detection of CMV viremia was associated with mortality.

### Causes of Death in Children With Cytomegalovirus Viremia

Causes of death for all children who died are shown in Table 3. The most frequent cause of death was pneumonia (n = 14), followed by sepsis (n = 10). Based on available data, no immediate cause of death was adjudicated as being caused by CMV.

**Table 3.** Causes of Death and CMV Viremia Among Children Starting NNRTI-Based ART in Uganda and Zimbabwe

Cause of Death^a^	N			Proportion CMV Viraemic^b^ at ART Initiation		
	Total	Uganda	Zimbabwe	Total	Uganda^c^	Zimbabwe^c^
Pneumonia	14	8	6	12/14 (86%)	6/8 (75%)	6/6 (100%)
Sepsis	10	5	5	7/10 (67%)	2/5 (40%)	5/5 (100%)
Cardiovascular disease (stroke, pulmonary hypertension, other cardiovascular causes)	6	2	4	5/6 (83%)	1/2 (50%)	4/4 (100%)
Malnutrition, wasting syndrome, diarrhea	5	4	1	2/5 (40%)	2/4 (50%)	0/1 (0%)
Tuberculosis	2	2	0	0/2 (0%)	0/2 (0%)	0/0 (0%)
HIV encephalopathy	1	1	0	1/1 (100%)	1/1 (100%)	0/0 (0%)
Malaria (*Plasmodium falciparum*)	1	1	0	1/1 (100%)	1/1 (100%)	0/0 (0%)
Meningitis	1	1	0	0/1 (0%)	0/1 (0%)	0/0 (0%)
Kaposi sarcoma	1	1	0	1/1 (100%)	1/1 (100%)	0/0 (0%)
Traumatic	1	1	0	0/1 (0%)	0/0 (0%)	0/0 (0%)
Unknown	4	3	1	1/4 (25%)	1/3 (33%)	0/1 (0%)

^a^Primary cause of death established via an independent expert review committee provided with a clinical narrative and available laboratory and radiology results.

^b^Excluding those detectable but not quantifiable.

^c^Percentage given is the proportion of viremic deaths/deaths per country.

### Cytomegalovirus Is Not Associated With Immune Activation

We finally explored the hypothesis that CMV reactivation is associated with T-cell activation, which could provide a plausible pathway to mortality. From 256 children who were also enrolled in the immunology substudy, immunophenotyping data at ART initiation was available for 48 Ugandan children. We found no evidence of difference in the proportions of CD4^+^ T cells that were naive (*P* = .83), activated (*P* = .65), or proliferating (*P* = 1.00) among this subset of children with detectable CMV at ART initiation (n = 12), compared to those without detectable CMV (n = 36).

## DISCUSSION

Mortality is high among children with HIV in the first months after starting ART [5]. Defining the risk factors for early mortality may inform novel interventions to improve outcomes in advanced HIV disease. We evaluated CMV viremia among children in Uganda and Zimbabwe and have 3 main findings. First, CMV viremia was common among children with HIV at ART initiation, particularly when sensitive assays were used, since most children had low-level viremia. Second, CMV viremia was associated with HIV disease severity; viremia gradually declined after ART initiation. Third, CMV viremia was independently associated with higher mortality after ART initiation, although deaths were not evidently due to CMV end-organ disease. Collectively, these findings show that CMV may be a viable target to improve survival in children with HIV in sub-Saharan Africa.

Cytomegalovirus viremia was common at ART initiation, consistent with other studies demonstrating a high prevalence of CMV viremia in children with HIV [19–21]. Cytomegalovirus viremia prevalence differed by country and assay type. In Uganda, one-quarter of children had CMV viremia at ART initiation; in Zimbabwe, where a more sensitive assay was used, three-quarters of children had CMV viremia. In viremic children, CMV viral loads were mostly low, with 10% of children in both countries having high-level viremia (>1000 copies/mL). Independent predictors of baseline CMV viremia were WHO stage, age, CD4%, and HIV RNA. These findings likely reflect the early-life acquisition of CMV in sub-Saharan Africa [6], with subsequent subclinical reactivation in the context of severe immunosuppression. HIV itself has been associated with subclinical reactivation of CMV in the vascular endothelium [14, 22, 23] and at mucosal surfaces, with detection in genital secretions, saliva, urine, and the gastrointestinal tract [14, 24].

There is growing evidence supporting a relationship between CMV viremia and mortality in adults initiating ART [25, 26], particularly among those with cryptococcal and tuberculous meningitis [27, 28]. In children recruited to the ARROW trial, we found that CMV viremia at the time of ART initiation was strongly associated with mortality in both countries independently of HIV RNA, immunosuppression, and biomarkers of immune activation. Overall, two-thirds of children who died had detectable CMV viremia at ART initiation. In Uganda, detectable CMV viremia was associated with a 3-fold increase in mortality. In Zimbabwe, we observed a 2-fold increase in mortality for every log_10_ higher CMV viral load. Our findings complement those from a pediatric study in Kenya, which found that CMV viremia (defined as ≥1000 IU/mL) at HIV diagnosis doubled the risk of mortality at 6 months and that increasing CMV viral loads were associated with a greater risk of mortality [20]. Similar to our findings, CMV viremia in Kenya was positively associated with HIV RNA and negatively associated with age.

We explored causes of mortality as adjudicated by an independent expert review committee, who had access to clinical narratives. Most children died of infections, with pneumonia and sepsis the most common causes of death. It is possible that some pneumonias were caused by CMV; however, CMV viremia was equally common among children who died from other causes, such as cardiovascular disease. Overall, there was no clear evidence of CMV end-organ disease in this cohort, and it seems more plausible that CMV acted as a co-factor in driving mortality.

There has been renewed interest in the indirect effects of CMV on immune function [29–31]. Cytomegalovirus drives a high-magnitude immune response: It has been estimated that around 10% of all CD4^+^ and CD8^+^ cells in memory compartments are CMV specific [32]. Controlling CMV infection is therefore a resource-intense immune process. Cytomegalovirus infection accelerates CD8^+^ T-cell maturation, leading to immunosenescence and cellular exhaustion [14]. HIV-associated CD4^+^ T-cell loss and dysfunction may hinder control of CMV by failing to provide help for CMV-specific CD8^+^ T-cell responses, resulting in inflammation and immunosenescence [14]. T-cell activation is a stronger predictor of mortality than HIV RNA [33], which might provide 1 mechanism by which CMV accelerates disease progression and risk of death. In a small subgroup of children, we found no evidence that those with CMV viremia had more activated or proliferating CD4^+^ cells, although this analysis was likely underpowered to detect differences in cellular phenotypes. Further work is needed to evaluate the underlying mechanism, whereby CMV accelerates HIV disease progression. Cytomegalovirus viremia might be associated with an increased risk of acquiring other co-infections; for example, associations between CMV viremia and tuberculosis have been reported in both children and adults [34, 35].

This study has strengths and weaknesses. We used data from a large cohort initiating ART recruited from 2 countries with a high HIV prevalence. We leveraged longitudinal outcome data, and the participants in this study were well phenotyped, providing rich covariate data. However, we cannot exclude residual confounding. We used molecular methods to detect CMV rather than relying on serology. However, different platforms were used to quantify CMV DNA in Uganda and Zimbabwe, with varying sensitivities. This rendered direct comparisons across countries challenging, and because of the different limits of detection, we treated these as 2 separate cohorts in the primary analysis. The higher CMV prevalence in Zimbabwe, compared with Uganda, likely reflected differences in assay sensitivity, although it is plausible that the true prevalence of CMV differed between settings. A secondary analysis that combined data from both countries showed similar results, confirming that baseline CMV viremia is associated with elevated mortality across settings. We only measured CMV in blood, although it is possible that CMV reactivation was apparent in other compartments. We did not directly collect data on CMV end-organ disease, limiting our evaluations of the causes of mortality. We included IL-6 and CD4 in models as covariates and hence estimated the direct effect of baseline CMV viremia after accounting for their effects (as well as those of age and HIV RNA), but it is possible that they may be on the causal pathway to mortality, ie may be mediators of the total effect of CMV viremia rather than confounders. Further mechanistic studies are required to understand the pathogenic pathways through which CMV may influence mortality. Nevertheless, the total effects of CMV viremia would be expected to be greater than the direct effects, so our conclusions still hold. Finally, the ART initiated in children in this study was NNRTI based; however, practice has evolved and now integrase inhibitor-based regimens are used first-line treatment [36]. Children in this study commenced ART at older ages with advanced HIV disease; it is possible that mortality effects differ in children who start ART at younger ages.

In conclusion, CMV was independently associated with mortality among children with HIV commencing ART in sub-Saharan Africa. There is growing interest in the role of CMV in driving mortality: A recent trial showed some evidence of reductions in mortality among infants hospitalized with HIV-associated pneumonia who were randomized to a 15-day course of empirical valganciclovir [37]; however, further trials are needed to evaluate whether all children with HIV would benefit from anti-CMV strategies. Alongside interventions to ensure earlier diagnosis and treatment for children with advanced HIV disease, we therefore propose that strategies to suppress CMV viremia at the time of initiating ART should be evaluated for their impact on mortality.

## Supplementary Material

ciag354_Supplementary_Data

## Supplementary Data

Supplementary materials are available at *Clinical Infectious Diseases* online. Consisting of data provided by the authors to benefit the reader, the posted materials are not copyedited and are the sole responsibility of the authors, so questions or comments should be addressed to the corresponding author.
