# Supplementary material for: Cytomegalovirus Viremia Is Associated With Mortality Among Children With HIV Starting Antiretroviral Therapy in Sub-Saharan Africa
Source: Clin Infect Dis. 2026 Jun 9;83(4):e807–15. doi: 10.1093/cid/ciag354 (PMC13715984; doi:10.1093/cid/ciag354)
Supplement: Supplementary file 1 [file ciag354_supplementary_data.docx]

**Supplementary Figure 1: Flow diagram of participants included in this analysis**

**Supplementary Table 1: Baseline characteristics of children starting NNRTI-based ART in Uganda and Zimbabwe by country**

| **Characteristic** | **Overall**  **(N=498)** | **Uganda**  **(N=336)** | **Harare**  **(N=162)** | **p** |
| --- | --- | --- | --- | --- |
| Site | | | | N/A |
| Uganda Entebbe (non-urban) | 57 (11.5%) | 57 (17.0%) | 0 |  |
| Uganda JCRC (urban) | 142 (28.5%) | 142 (42.3%) | 0 |  |
| Uganda PIDC (urban) | 137 (27.5%) | 137 (40.8%) | 0 |  |
| Zimbabwe Harare (urban) | 162 (32.5%) | 0 | 162 (100%) |  |
| In immunology sub-study | 256 (51.4%) | 173 (51.5%) | 83 (51.2%) | 0.96 |
| Male sex | 239 (47.8%) | 156 (46.4%) | 83 (51.2%) | 0.32 |
| Age, years; median (IQR) | 6.3 (2.3, 9.6) | 5.2 (2.2, 9.6) | 7.5 (3.9, 9.7) | 0.003 |
| HIV viral load, copies/mL; median (IQR) | 238723  (81090, 641190) | 237619  (75897, 769330) | 243136  (114067, 453534) | 0.52 |
| CD4%; median (IQR) | 11 (6, 17) | 12 (7, 18) | 10 (5, 14) | 0.006 |
| WHO clinical stage | | | | <0.001 |
| 1 | 9 (1.8%) | 9 (2.7%) | 0 |  |
| 2 | 147 (29.5%) | 125 (37.2%) | 22 (13.6%) |  |
| 3 | 264 (53.0%) | 148 (44.0%) | 116 (71.6%) |  |
| 4 | 78 (15.7%) | 54 (16.1%) | 24 (14.8%) |  |
| Detectable CMV at baseline* | 208 (41.8%) | 89 (26.5%) | 119 (73.5%) | <0.001 |
| Plasma CRP, mg/L; median (IQR) | 4.6 (1.4, 14.6) | 4.9 (1.5, 14.8) | 3.8 (1.4, 13.8) | 0.43 |
| Plasma IL-6, pg/mL; median (IQR) | 6.2 (4.7, 9.5) | 6.2 (4.6, 9.5) | 6.1 (4.9, 9.6) | 0.50 |

* Different assay thresholds (120 copies/ml in Uganda, 20 copies/ml in Harare).

JCRC = Joint Clinical Research Centre; PIDC = Paediatric Infectious Disease Clinic, Mulago Hospital

**Supplementary Table 2 Independent associations between CMV viraemia at ART initiation (log_10_ transformed) and other baseline covariates**

| **Covariate** | **Effect on log_10_ baseline CMV VL** | **95% CI** | **P** |
| --- | --- | --- | --- |
| Zimbabwe vs Uganda | 0.48 | (+0.25, +0.71) | <0.001 |
| Female vs male | 0.03 | (-0.18, +0.24) | 0.76 |
| WHO stage 3 vs 2 | 0.08 | (-0.17, +0.34) | 0.63 |
| WHO stage 4 vs 2 | 0.53 | (+0.21, +0.85) | 0.001 |
| Immunology substudy | -0.09 | (-0.29, +0.12 | 0.41 |
| Per 5 years older | -0.15 | (-0.23, -0.08) | <0.001 |
| Per 5 CD4% higher | -0.44 | (-0.58, -0.29) | <0.001 |
| Per log10 HIV RNA higher | 0.39 | (+0.22, +0.56) | <0.001 |

Note: no additional independent effect of CRP (p=0.14), IL-6 (p=0.07), TNFa (p=0.45) or soluble CD14 (p=0.25) after inclusion of factors shown above.

**Supplementary Table 3: Associations between mortality and CMV viraemia among children starting NNRTI-based ART in Uganda and Zimbabwe not adjusting for IL-6**

|  | **Unadjusted HR (95%CI)** | **P** | **Adjusted* HR (95%CI)** | **P** | **Heterogeneity in effect by country** |
| --- | --- | --- | --- | --- | --- |
| **Primary analysis** | | | | | |
| **Detectable vs. not detectable using country specific assay thresholds** | | | | | |
| **Uganda** | 3.01 (1.09, 8.25) | 0.03 | 3.00 (1.06, 8.35) | 0.04 | - |
| **Zimbabwe** | 2.01 (0.23, 17.3) | 0.52 | 1.79 (0.20, 16.2) | 0.61 | P=0.68 |
| **Per log_10_ higher CMV (if detectable using country specific assay thresholds)** | | | | | |
| **Uganda** | 1.03 (0.48, 2.20) | 0.95 | 0.95 (0.41, 2.20) | 0.91 | - |
| **Zimbabwe** | 2.37 (1.52, 3.68) | <0.001 | 2.86 (1.64, 4.98) | <0.001 | P=0.02 |
| **Secondary analyses** | | | | | |
| **Above vs. below 120 copies/ml** | | | | | |
| **Uganda** | 3.08 (1.49, 6.38) | 0.002 | 2.54 (1.15, 5.63) | 0.02 | - |
| **Zimbabwe** | 2.38 (0.88, 6.43) | 0.09 | 2.29 (0.84, 6.26) | 0.16 | P=0.87 |
| **Combined** | 2.85 (1.59, 5.10) | <0.001 | 2.45 (1.31, 4.59) | 0.005 |  |
| **Above vs. below 1000 copies/ml** | | | | | |
| **Uganda** | 2.00 (0.76, 5.23) | 0.16 | 1.39 (0.48, 4.06) | 0.54 | - |
| **Zimbabwe** | 13.9 (5.33, 36.3) | <0.001 | 9.81 (3.68, 26.2) | <0.001 | P=0.006 |
| **Combined** | 4.59 (2.45, 8.60) | <0.001 | 3.62 (1.74, 7.54) | 0.001 | - |

* One Cox model fitted for all children, adjusted for participation in the immunology substudy, CD4, HIV viral load and age concentration at ART initiation, and including interaction between CMV variables and country (see Table 2 for main analyses, also adjusting for plasma IL6). HR=hazard ratio.
